# Comparison of waist-height ratio and other obesity indices in the prediction of diabetic peripheral neuropathy

**DOI:** 10.3389/fnut.2022.949315

**Published:** 2022-10-06

**Authors:** Yakubu Lawal, Rifkatu Mshelia-Reng, Special O. Omonua, Kenechukwu Odumodu, Ramatu Shuaibu, Ukamaka D. Itanyi, Amina I. Abubakar, Hadijat O. Kolade-Yunusa, Zumnan D. Songden, Caleb O. Ehusani, Olufemi Adediran, Felicia E. Anumah

**Affiliations:** College of Health Sciences, University of Abuja, Abuja, Nigeria

**Keywords:** central obesity, diabetes mellitus, peripheral neuropathy, waist-height ratio, waist circumference

## Abstract

**Background:**

Waist-height ratio (WHtR) is increasingly being studied as a simple and effective measure of central obesity. Reports have shown that WHtR is a better predictor of hypertension, diabetes, and cardiovascular diseases when compared to traditional obesity indices like body mass index (BMI), waist circumference (WC), and waist-hip ratio (WHR). This study is therefore aimed at comparing WHtR with other obesity indices in the prediction of peripheral neuropathy in persons with diabetes mellitus (DM).

**Methodology:**

One thousand and forty persons with DM were enrolled following consent. Relevant details of history were obtained, followed by physical examinations. Data were analyzed using IBM-SPSS version 23. Logistic regression was used to compare the odds ratio of obesity indices in the prediction of peripheral neuropathy. The level of significance used was *p* = 0.05.

**Results:**

Logistic regression showed that WHtR had the highest odds ratio (OR) for the prediction of “probable” diabetic peripheral neuropathy (OR 9.11, 95% CI 3.07–47.97, *p* = 0.002), followed by WC (OR 2.01, 95% CI 1.09–4.05, *p* = 0.004), and BMI (OR 1.26, 95% CI 1.00–3.99, *p* = 0.019) after correction for age; systemic hypertension; duration of DM; control of SBP, DBP, HbA1c, FPG, and 2HrPP.

**Conclusion:**

WHtR has the highest odds ratio in the prediction of “probable” diabetic peripheral neuropathy in both genders, followed by WC in the males and BMI in the females.

## Introduction

Waist-height ratio (WHtR) is one of the obesity indices that is used to measure central obesity. It is the ratio of waist circumference (WC) as the numerator and height as the denominator, all in the same units. Generally, it has been reported that WHtR is a more accurate measure of metabolically significant obesity than body mass index (BMI), WC, and waist-hip ratio (WHR) (1–4).

Similarly, other reports have shown that WHtR is one of the obesity indices that best predicts hypertension, diabetes, and cardiovascular diseases (1–7). However, fewer reports have shown similar predictions of peripheral neuropathy by obesity indices (8–10). In a similar vein, Bulum et al. (11) demonstrated that WHtR was better than WC in predicting diabetic nephropathy, another microvascular complication of diabetes mellitus (DM). It is therefore important to identify the obesity index with the highest predictive power for peripheral neuropathy. This will allow for early recognition of persons at risk of diabetic peripheral neuropathy and adoption of measures to prevent or delay the complication.

## Methodology

This multi-center cross-sectional study was aimed at comparing WHtR with other obesity indices in the prediction of diabetic peripheral neuropathy in North Central Nigeria. This study was carried out as part of routine clinical activities in multiple hospitals and in accordance with the 1964 Helsinki Declaration and its later amendments or comparable ethical standards. Inclusion criteria included persons with both type 1 and type 2 DM, while exclusion criteria included those who were not diagnosed as having DM. Consent was sought and obtained from the participants to allow the use of their clinical details with no identity disclosure. A total of 1040 participants were consecutively enrolled over the period of the study. Personal details and other relevant history were obtained from each participant, and age grouping was based on World Health Organization (WHO) age classification (12).

Height and weight were measured using a height-weight scale (RGZ-120 model, J Suhong Medicals company; Jiangsu, China; July 2017). With each participant standing straight with apposed feet, heels against the back board with neither footwear nor head cover, looking straight ahead, and the external auditory opening horizontally aligned with the lower orbital margin, the height was measured at the peak of inspiration to the nearest 0.1 cm. To measure each participant’s weight, he or she was asked to dress lightly, stand still without footwear on the scale, feet separated and parallel, looking straight ahead, and arms hanging by the sides. The weight was then measured to the nearest 0.5 kg. BMI in kg/m^2^ was then calculated by dividing weight in kilograms by the square of height in square meters (13). Then, WC was measured using a non-stretchable tape. With the examiner and an assistant standing by each side of a participant, the measuring tape was wrapped around each participant’s waist at the mid-point between the inferior border of the lowest rib and the superior border of the iliac crest, and parallel to the horizontal plane. The WC was then measured at the end of expiration to the nearest 0.1 cm (11). WHtR was calculated by dividing WC (in meters) by height (meters); a value of > 0.5 was considered as central obesity for both males and females (14).

Furthermore, Semmes-Weinstein 10 g monofilament was used to test for protective sensation. Following a test application to the forehead, the filament was applied perpendicular to the plantar surface of the 1st and 3rd toes and respective metatarsal heads with the participant’s eyes closed. Minimum pressure just enough for the filament to buckle was exerted within 2 s. Care was taken to avoid repeated contact, sliding, or application on corns, calluses, ulcers, or ischemic areas. The participant was asked to say: “Yes” on feeling the filament. The procedure was repeated twice at each site with at least one “mock” test with an irregular timing pattern to avoid anticipation of stimulus by the participants. “Probable” diabetic peripheral neuropathy was diagnosed if there was a failure to feel the sensation at two or more points on either foot (15, 16).

Vibration sensation was tested using a 128 Hz tuning fork, struck against the examiner’s palm to vibrate for at least 40 s. Following the test application to the participant’s forehead, the base of the tuning fork was applied to the bony prominence at the dorsum of the big toe just proximal to the nail bed with the participant’s eyes closed. He or she was then asked to acknowledge the vibration sensation, and then tell when it stopped following dampening by the examiner. This was repeated twice on each foot in addition to one “mock” application irregularly timed, to prevent stimulus anticipation. “Probable” diabetic peripheral neuropathy was diagnosed when there was failure to sense vibration or acknowledge when it stopped, in at least two sites on either foot (15, 16).

To examine the ankle reflex, each participant was asked to lie supine, hip slightly externally rotated, foot gently dorsiflexed, then the Achilles tendon was tapped with a reflex hammer. Jendrassik maneuver (interlocking and pulling flexed fingers) was used to reinforce the reflex if it was not noticed during the first attempt. Participants with absent plantar flexion and reduced or absent reflex contraction of gastrocnemii were diagnosed as having “probable” diabetic peripheral neuropathy (15, 16).

Microsoft Excel was used for data entry, and then statistical analysis was done using IBM SPSS version 23 (IBM SPSS Co., Ltd., New York, USA; March 4, 2015). Logistic regression was used to compare the odds ratio of obesity indices in the prediction of peripheral neuropathy after correcting for other determinants or confounding factors. The significance level used was *p* < 0.05.

## Results

The aim of this study was to compare the WHtR with other obesity indices in the prediction of diabetic peripheral neuropathy. The total number of participants was 1,040, i.e., 432 males and 608 females in the ratio of 1.0–1.4, comprising 1,002 and 38 persons with type 2 and type 1 DM, respectively. The mean age (SD) of the participants was 52.91 (11.82) years with a range of 15–99 years. The prevalence of diabetic peripheral neuropathy was significantly higher among participants with obesity by WHtR (66%, *p* < 0.001), by WC (60%, *p* < 0.001), and by BMI (26.8%, *p* < 0.001) (Table 1).

**TABLE 1:** Prevalence of peripheral neuropathy in persons with obesity (by different obesity indices).

Obesity indices	Normal	P.Neuro	Chi square	*p*
BMI	6.7%	26.8%	14.17	<0.001
WC	13.6%	60.1%	45.39	<0.001
WHtR	15.5%	66.0%	51.67	<0.001

BMI, body mass index; WC, waist circumference; WHtR, waist-height ratio, *p* = level of significance, normal = no peripheral neuropathy, P.Neuro = peripheral neuropathy.

Logistic regression showed that WHtR had the highest odds ratio (OR) for the prediction of peripheral neuropathy (OR 9.11, 95% CI 3.07–47.97, *p* = 0.002), followed by WC (OR 2.01, 95% CI 1.09–4.05, *p* = 0.004), and BMI (OR 1.26, 95% CI 1.00–3.99, *p* = 0.019) after correction for age; systemic hypertension; duration of DM; control of SBP, DBP, HbA1c, FPG, and 2 HrPP. Following gender sub-analysis, WHtR maintained the highest OR in the males (OR 5.00, 95% CI 2.02–11.37, *p* = 0.007), followed by WC (OR 1.08, 95% CI 1.02–2.14, *p* = 0.015), and BMI (OR 1.05, 95% CI 0.91–1.81, *p* = 0.101). Among the female participants, WHtR also had the highest OR in the prediction of peripheral neuropathy (OR 14.43, 95% CI 5.15–127.21, *p* = 0.005), followed, however, by BMI (OR 2.96, 95% CI 1.92–4.01, *p* = 0.019), then WC (OR 2.00, 95% CI 1.05–3.04, *p* = 0.037) (Table 2).

**TABLE 2:** Logistic regression showing odds ratio of obesity indices for peripheral neuropathy.

	B	S.E.	Wald	df	p	Exp(B)	95% C.I. for Exp(B)	
							Lower	Upper
**All Participants**							
BMI	0.043	0.018	5.534	1	0.019	1.258	1.004	3.993
WC	0.014	0.015	13.860	1	0.004	2.014	1.085	4.045
WHtR	2.210	2.478	26.795	1	0.002	9.112	3.071	47.966
**Males**								
BMI	0.049	0.030	2.689	1	0.101	1.052	0.908	1.810
WC	0.073	0.030	5.972	1	0.015	1.076	1.015	2.140
WHtR	7.626	5.131	2.209	1	0.007	5.002	2.017	11.369
**Females**								
BMI	0.037	0.024	2.427	1	0.019	2.963	1.919	4.010
WC	0.014	0.022	0.033	1	0.037	1.996	1.053	3.041
WHtR	4.945	3.476	2.023	1	0.005	14.429	5.154	127.210

BMI, body mass index; WC, waist circumference; WHtR, waist-height ratio; B, coefficient of regression; SE, standard of error; df, degree of freedom; *p*, level of significance; exp(B), odds ratio.

A sub-analysis for type 2 DM showed that WHtR had the highest odds ratio for peripheral neuropathy (OR 22.36, 95% CI 3.15–331.77, *p* = 0.023), followed by WC (OR 3.00, 95% CI 1.97–10.04, *p* = 0.032) after correction for age; systemic hypertension; duration of DM; control of SBP, DBP, HbA1c, FPG, and 2 HrPP. However, none of the obesity indices were significant predictors of peripheral neuropathy in a sub-analysis for persons with type 1 DM (Table 3).

**TABLE 3:** Logistic regression showing odds ratio of obesity indices for peripheral neuropathy in persons with type 1 and type 2 DM.

	B	S.E.	Wald	df	p	Exp(B)	95% C.I. for Exp(B)	
							Lower	Upper
**Type 2 DM**							
BMI	–0.051	0.019	6.982	1	0.098	0.950	0.915	0.987
WC	0.003	0.016	0.045	1	0.032	3.003	1.972	10.035
WHtR	3.107	2.550	1.484	1	0.023	22.357	3.151	331.771
**Type 1 DM**							
BMI	–0.302	0.162	3.471	1	0.062	0.739	0.538	1.016
WC	0.144	0.079	3.340	1	0.068	1.155	0.990	1.349
WHtR	–1.411	11.371	0.015	1	0.901	0.244	0.000	1.746

BMI, body mass index; WC, waist circumference; WHtR, waist-height ratio; *p*, level of significance; B, coefficient of regression; SE, standard of error; df, degree of freedom; exp(B), odds ratio.

Following sub-analysis based on age groups, WHtR had the highest odds ratio for the prediction of peripheral neuropathy, followed by WC, in the middle age and the elderly age groups after correction for age; systemic hypertension; duration of DM; control of SBP, DBP, HbA1c, FPG, and 2 HrPP. However, in the young age group, none of the obesity indices was a significant predictor of diabetic peripheral neuropathy (Table 4).

**TABLE 4:** Logistic regression showing odds ratio of obesity indices for peripheral neuropathy by age.

	B	S.E.	Wald	df	p	Exp(B)	95% C.I. for Exp(B)	
							Lower	Upper
**The young (<44 years)**						
BMI	–0.068	0.042	2.666	1	0.102	0.934	0.861	1.014
WC	0.036	0.028	1.667	1	0.197	1.036	0.982	1.094
WHtR	0.177	3.869	0.002	1	0.964	0.838	0.000	1647.130
**The middle age (44–59 years)**						
BMI	–0.024	0.027	0.779	1	0.377	0.976	0.925	1.030
WC	0.015	0.024	0.044	1	0.033	5.995	1.949	11.043
WHtR	3.257	4.009	0.660	1	0.017	25.976	5.010	67.203
**The elderly (=60 years)**						
BMI	–0.045	0.036	1.541	1	0.214	0.956	0.891	1.026
WC	0.046	0.035	1.712	1	0.041	3.047	1.977	11.122
WHtR	2.148	5.878	0.134	1	0.015	5.117	3.000	13.103

BMI, body mass index; WC, waist circumference; WHtR, waist-height ratio; *p*, level of significance; B, coefficient of regression; SE, standard of error; df, degree of freedom; exp(B), odds ratio.

## Discussion

This study was aimed at comparing WHtR with other obesity indices in the prediction of peripheral neuropathy among persons with DM. The prevalence of peripheral neuropathy was higher among participants with obesity than those without obesity and it was also higher in WHtR-defined obesity than in WC- and BMI-defined obesity. This may mean that WHtR has a higher predictive power for peripheral neuropathy compared to WC and BMI. Further analysis using logistic regression showed that WHtR had the highest odds ratio in the prediction of peripheral neuropathy compared to WC and BMI. Though, some studies have reported the significant abilities of obesity indices to predict cardiovascular diseases (1, 2, 5), with some reports demonstrating that WHtR had higher predictive power than WC and BMI (3, 4), very few studies have ventured into the predictive abilities of these obesity indices for peripheral neuropathy (8, 9). However, Bulum et al. (11) reported that WHtR was more accurate than WC in predicting diabetic nephropathy, which is also a microvascular complication of DM.

Following gender-based sub-analysis, WHtR was found to have the highest odds ratio in the prediction of peripheral neuropathy in both the male and female participants after correction for age; systemic hypertension; duration of DM; control of SBP, DBP, HbA1c, FPG, and 2 HrPP. However, WC had a higher predictive ability than BMI in the males, while BMI was more predictive of peripheral neuropathy than WC in the female participants. There was no similar report found that showed this gender difference in the prediction of peripheral neuropathy by these obesity indices. However, with regard to the prediction of cardiovascular diseases, Heffron et al. (5) showed that BMI had a higher predictive ability in women than in men. Further sub-analysis based on age groups showed a similar pattern of the higher odds ratio of WHtR than WC and BMI in the prediction of diabetic peripheral neuropathy among the middle-aged and the elderly. The young age group did not show a significant prediction of diabetic peripheral neuropathy by the obesity indices. This may be explained by the larger ratio of type 1 DM in the younger age group. Obesity is not a known risk factor for type 1 DM, and obesity prevalence is less in the younger age group, making it unlikely for obesity indices to be a significant predictor of diabetic peripheral neuropathy. This was further expounded by a sub-analysis based on types of diabetes, where WHtR and WC were significant predictors of peripheral neuropathy among persons with type 2 DM, while none of the obesity indices was a significant predictor of peripheral neuropathy among persons with type 1 DM.

The implication of our finding is that WHtR may be more reliable as a predictor of diabetic peripheral neuropathy across the gender divide. Additionally, the fact that WHtR is a simple, non-invasive, yet effective measure of obesity should encourage its use among clinicians, with a possible increased detection of those at risk of diabetic peripheral neuropathy leading to early adoption of preventive strategies. Though the results of this study may be generalizable, however, ethnic and racial differences in the cut-offs of obesity indices may limit this generalization. The limitations of this study include the non-application of the Michigan Neuropathy Screening Instrument (MNSI) questionnaire, and the non-availability of nerve conduction studies, a gold standard for the diagnosis of peripheral neuropathy.

## Conclusion

WHtR is a stronger predictor of diabetic peripheral neuropathy than WC and BMI in middle-aged, elderly, male, and female persons with type 2 DM. This was followed by waist circumference in the males and body mass index in the females. Clinicians are, therefore, encouraged to routinely use WHtR as a simple and effective tool to measure central obesity and assess the risk of developing diabetic peripheral neuropathy.

## Data availability statement

The raw data supporting the conclusions of this article will be made available by the authors, without undue reservation.

## Ethics statement

Ethical review and approval was not required for the study on human participants in accordance with the local legislation and institutional requirements. Written informed consent for participation was not required for this study in accordance with the national legislation and the institutional requirements.

## Author contributions

YL, RM-R, SO, KO, and RS involved in the conceptualization, data collection, analysis, drafting, and final approval of the manuscript. UI, AA, HK-Y, ZS, and CE involved in the data collection, analysis, proofreading, and final approval of the manuscript. FA and OA involved in the conceptualization, data collection, analysis, editing, and final approval of the manuscript. All authors contributed to the article and approved the submitted version.

## References

[1] Ononamadu CJ Ezekwesili CN Onyeukwu OF Umeoguaju UF Ezeigwe OC Ihegboro GO. Comparative analysis of anthropometric indices of obesity as correlates and potential predictors of risk for hypertension and prehypertension in a population in Nigeria. *Cardiovasc J Afr.* (2017) 28:92–9. 10.5830/CVJA-2016-061 27701484 PMC5488060

[2] Yeboah K Puplampu P Yorke E Antwi DA Gyan B Amoah AG. Body composition and ankle-brachial index in Ghanaians with asymptomatic peripheral arterial disease in a tertiary hospital. *BMC Obes.* (2016) 3:27. 10.1186/s40608-016-0107-3 27239322 PMC4866025

[3] Moosaie F Fatemi Abhari SM Deravi N Karimi Behnagh A Esteghamati S Dehghani Firouzabadi F Waist-to-height ratio is a more accurate tool for predicting hypertension than waist-to-hip circumference and BMI in patients with type 2 diabetes: a prospective study. *Front Public Health.* (2021) 9:726288. 10.3389/fpubh.2021.726288 34692623 PMC8529190

[4] Lawal Y Bello F Anumah FE Bakari AG. Waist-height ratio: how well does it predict glucose intolerance and systemic hypertension? *Diabetes Res Clin Pract.* (2019) 158:107925. 10.1016/j.diabres.2019.107925 31715203

[5] Heffron SP Dwivedi A Rockman CB Xia Y Guo Y Zhong J Body mass index and peripheral artery disease. *J Atherosclerosis.* (2020) 292:31–6. 10.1016/j.atherosclerosis.2019.10.017 31739257 PMC6981229

[6] Vikram NK Latifi AN Misra A Luthra K Bhatt SP Guleria R Waist-to-height ratio compared to standard obesity measures as predictor of cardiometabolic risk factors in Asian Indians in North India. *Metab Syndr Relat Disord.* (2016) 14:492–9. 10.1089/met.2016.0041 27740885

[7] Ferreira-Hermosillo A Ibarra-Salce R Rodríguez-Malacara J Molina-Ayala MA. Comparison of indirect markers of insulin resistance in adult patients with double diabetes. *BMC Endocr Disord.* (2020) 20:87. 10.1186/s12902-020-00570-z 32539854 PMC7296956

[8] Franceschi R Mozzillo E Di Candia F Rosanio FM Leonardi L Liguori A A systematic review of the prevalence, risk factors and screening tools for autonomic and diabetic peripheral neuropathy in children, adolescents and young adults with type 1 diabetes. *Acta Diabetol.* (2022) 59:293–308. 10.1007/s00592-022-01850-x 35089443

[9] Christensen DH Knudsen ST Gylfadottir SS Christensen LB Nielsen JS Beck-Nielsen H Metabolic factors, lifestyle habits, and possible polyneuropathy in early type 2 diabetes: a nationwide study of 5,249 Patients in the Danish centre for strategic research in type 2 diabetes (DD2) cohort. *Diabetes Care.* (2020) 43:1266–75. 10.2337/dc19-2277 32295810

[10] Sauder KA Stafford JM Mayer-Davis EJ Jensen ET Saydah S Mottl A Co-occurrence of early diabetes-related complications in adolescents and young adults with type 1 diabetes: an observational cohort study. *Lancet Child Adolesc Health.* (2019) 3:35–43. 10.1016/S2352-4642(18)30309-2 30409691 PMC6295346

[11] Bulum T Blaslov K Duvnjak L. The use of anthropometric measurements of obesity in prediction of microvascular complications in obese type 2 diabetic patients. *Acta Clin Croat.* (2016) 55:217–23. 10.20471/acc.2016.55.02.06 28394108

[12] Dyussenbayev A. Age periods of human life. *Adv Soc Sci Res J.* (2017) 4:6. 10.14738/assrj.46.2924

[13] World Health Organization. *WHO STEPS Surveillance Manual 2017: The WHO STEPwise Approach to Chronic Disease Risk Factor Surveillance.* Geneva: World Health Organization (2005).

[14] Browning LM Hsieh SD Ashwell M. A systematic review of waist-to-height ratio as a screening tool for the prediction of cardiovascular disease and diabetes: 0⋅5 could be a suitable global boundary value. *Nutr Res Rev.* (2010) 23:247–69. 10.1017/S0954422410000144 20819243

[15] Jayaprakash P Bhansali A Bhansali S Dutta P Anantharaman R Shanmugasundar G Validation of bedside methods in evaluation of diabetic peripheral neuropathy. *Indian J Med Res.* (2011) 133:645–9. 21727664 PMC3135993

[16] Lanting SM Spink MJ Tehan PE Vickers S Casey SL Chuter VH. Non-invasive assessment of vibration perception and protective sensation in people with diabetes mellitus: inter- and intra-rater reliability. *J Foot Ankle Res.* (2020) 13:3. 10.1186/s13047-020-0371-9 31988664 PMC6966840

